# The Use of Aminated Yerba Mate Biomass for the Removal of Anionic Dyes from Aqueous Solutions

**DOI:** 10.3390/ma19091722

**Published:** 2026-04-23

**Authors:** Tomasz Jóźwiak, Urszula Filipkowska, Przemysław Charubin

**Affiliations:** Department of Environmental Engineering, University of Warmia and Mazury in Olsztyn, Warszawska St. 117a, 10-957 Olsztyn, Poland; urszula.filipkowska@uwm.edu.pl (U.F.); przemyslaw.charubin@student.uwm.edu.pl (P.C.)

**Keywords:** yerba mate, biomass, unconventional adsorbent, amination, cationization, chemical modification, adsorption, dyes, Reactive Black 5, Reactive Yellow 84

## Abstract

In line with circular economy principles, raw spent yerba mate (*Ilex paraguariensis*) waste (YMs) was transformed into a high-value aminated adsorbent (AYMs) for the removal of anionic dyes, namely Reactive Black 5 (RB5) and Reactive Yellow 84 (RY84). The modification involved a two-step process using epichlorohydrin and aqueous ammonia, and the adsorbents were characterized via FTIR, BET, C/N elemental analysis, and pH_PZC_. Batch experiments evaluated pH effects, kinetics (PFO, PSO, and intraparticle diffusion), and equilibrium isotherm analysis (single- and dual-site Langmuir models and Freundlich models). The results confirmed successful functionalization of the biomass with amino groups, shifting the point of zero charge (pH_PZC_) from 4.74 (YMs) to 8.73 (AYMs). The optimal adsorption pH was 2.0 for YMs and 3.0 for AYMs. Kinetic data were best described by the pseudo-second-order model, while equilibrium data followed the dual-site Langmuir model, indicating energetic heterogeneity of the AYMs surface. The maximum adsorption capacity of AYMs reached 62.81 mg·g^−1^ for RB5 and 61.78 mg·g^−1^ for RY84, representing a fivefold and threefold increase over the YMs, respectively. These findings demonstrate that AYMs is a high-performance, sustainable alternative to commercial activated carbons, providing a scalable waste-to-value solution for industrial effluent treatment.

## 1. Introduction

Post-production synthetic dyes are complex organic compounds with a high degree of saturation by chromophoric and auxochromic groups, which give them intense color and the ability to bond permanently to substrates. Currently, over 1 million tons of dyes, representing more than 100,000 different chemical structures, are produced annually worldwide [1]. Reactive dyes make up the largest group by production volume (approximately 32–35%), primarily used in the textile industry, which consumes about 70% of the total global supply of these substances [2]. The remainder is used in the paper, leather, cosmetic, and food industries. Due to the specifics of technological processes, especially in the textile sector, dyes do not bind completely to fabric. For anionic dyes (acid and reactive), process losses can reach up to 50% of the colorant mass, resulting in industrial effluents with an extremely high dye load [3]. Inadequate wastewater management poses a significant risk of discharging colored effluents into the natural environment, with local water bodies being most vulnerable.

Synthetic colorants remain perceptible to the naked eye in aquatic systems even at highly dilute concentrations, specifically those under 1 mg·L^−1^ [4]. Intense coloration of water bodies drastically limits sunlight penetration into deeper layers, inhibiting photosynthesis, leading to oxygen depletion, and, in extreme cases, resulting in anaerobic conditions [5]. Furthermore, many artificial colorants and their breakdown intermediates are recognized as hazardous substances with significant carcinogenic potential for both aquatic and terrestrial organisms. A frequent consequence of colorant pollution is the partial or total degradation of local aquatic ecosystems [6].

The imperative to protect the natural environment requires the implementation of Best Available Techniques (BAT) for effluent decolorization. Contemporary environmental engineering offers various dye removal methods, which can be categorized as biological or physicochemical processes. Biological decolorization methods, typically based on activated sludge or biofilm technologies, are relatively cost-effective; however, they have limited efficiency owing to the significant biological recalcitrance of most synthetic dyes [7]. Most reactive dyes are engineered to be highly stable and resistant to oxidation, making biological processes time-consuming [8].

Physicochemical methods provide an alternative to biological treatments. For example, coagulation and flocculation allow rapid dye removal but generate substantial amounts of chemical sludge, which is costly to dispose of [9]. Membrane techniques, such as reverse osmosis, achieve decolorization efficiencies exceeding 99%, yet they face challenges like membrane fouling and high capital costs [10]. Additionally, Advanced Oxidation Processes are not effective for all dye types and may produce intermediate products with toxicity often exceeding that of the parent compounds [11].

The adsorption process is an efficient alternative to these methods. It involves the accumulation of pollutant molecules (adsorbate) on the surface or within the pores of a solid material (adsorbent). The main advantages of this method include simple system design and the absence of toxic by-product generation. The yield of the sorption process relies on a complex interplay between the aqueous medium’s conditions (primarily pH and temperature) and the functional features of the solid adsorbent [12]. Activated carbon, derived from fossil coals (such as hard coal or lignite) or plant-based precursors, is the most commonly used conventional adsorbent [13]. The adsorption properties of activated carbon are primarily due to its porosity and large specific surface area, often exceeding 1000 m^2^·g^−1^ [14]. Spent activated carbon can be regenerated through re-carbonization and activation [15]. Nevertheless, the substantial energy requirements associated with both the synthesis and thermal restoration of activated carbon contribute to high market prices, which can limit its widespread application.

Currently, researchers are focusing on the potential to obtain adsorbents from agri-food waste products, such as non-edible parts of crops. These materials have near-zero market value, and their use aligns with sustainable development strategies. To date, the literature has described the adsorption potential of numerous plant-based waste products, including cereal husks, fruit and vegetable peels, and tree leaves [16,17,18,19].

In their previous research, the authors demonstrated the adsorption potential of spent yerba mate (*Ilex paraguariensis)* [20]. The choice of this raw material as an adsorbent was driven by the significant waste generated from yerba mate preparation, a result of its increasing global popularity [21]. Additionally, spent yerba mate exhibits morphological diversity, providing a rich profile of functional groups derived from cellulose, hemicellulose, and lignin. The results of these studies confirmed high efficiency in removing cationic dyes [20]. However, a significant drawback of this adsorbent—consistent with other unmodified plant biomass materials—was its generally low adsorption capacity for anionic dyes [20]. This limitation arises because lignocellulosic biomass typically has an acidic character. Within the standard pH range of wastewater, the surface of plant materials usually carries a negative charge [22]. This electrostatic barrier represents a major obstacle to efficient anionic dye removal for unmodified materials.

A solution to the low adsorption efficiency of plant biomass is the chemical modification of the material via amination, which leads to its cationization. Amination involves a reaction with an aminating agent, introducing additional amino groups (-NH_2_) into the chemical structure of the lignocellulosic matrix [23]. In both acidic and neutral environments, these groups undergo ionization (–NH_3_^+^), imparting a positive charge to the aminated adsorbent. These protonated functional groups facilitate the formation of strong ionic bonds with dye anions, thereby significantly increasing the adsorption capacity for anionic pollutants, including reactive dyes.

While the effectiveness of this strategy has been confirmed for various substrates, a critical evaluation of the literature reveals significant performance disparities. Raw lignocellulosic residues consistently exhibit poor adsorption capacities for anionic dyes, often failing to exceed 15 mg·g^−1^ [20,24,25,26,27]. For instance, raw sunflower biomass and cotton fibers achieve capacities as low as 1.10 mg·g^−1^ [28] and 2.74 mg·g^−1^ [24], respectively. Chemical amination addresses these quantitative limitations, yet the efficiency of the modified materials varies greatly. Reported capacities for aminated cotton fibers reach approximately 36.77 mg·g^−1^ [24], whereas more efficient aminated residues, such as goldenrod biomass (71.30 mg·g^−1^) [27] or buckwheat hulls (85.18 mg·g^−1^) [25], demonstrate much higher potential. Despite these advancements, there is currently no data on the amination of *Ilex paraguariensis*. This represents a notable gap, especially as yerba mate’s unique structural profile—containing high lignin (25–30%) and protein (4–12%) content—may provide a more robust chemical matrix for amino group stabilization than previously studied agricultural wastes.

While our previous work [20] identified a significant efficiency gap in the removal of anionic pollutants by raw spent yerba mate, the present study addresses this limitation through targeted chemical amination. Unlike the earlier research, which focused on the baseline potential of the raw biomass, this work provides a direct, systematic comparison between unmodified spent yerba mate (YMs) and aminated spent yerba mate (AYMs), specifically targeting highly stable reactive dyes (Reactive Black 5 and Reactive Yellow 84). The aim of this research was to characterize the modified adsorbent (FTIR, BET, elemental analysis: C, N, pH_PZC_) and evaluate its performance through pH effect studies, adsorption kinetics (PFO, PSO, and intraparticle diffusion models), and equilibrium isotherms (single- and dual-site Langmuir models and Freundlich models). This comprehensive approach allows for a clear demonstration of how chemical functionalization upgrades yerba mate waste into a high-performance, specialized adsorbent.

## 2. Materials

### 2.1. Spent Yerba Mate Biomass

Paraguayan holly (*Ilex paraguariensis*) biomass was used as the raw material for adsorbent preparation. The research material consisted of the commercial product “YERBA MATE Amanda Elaborada” (La Cachuera S.A., Apóstoles, Argentina), purchased from a retail chain (Auchan, Olsztyn, Poland). The raw material included ground leaves and stems. To simulate the generation of typical waste from yerba mate biomass processing, it underwent an aqueous extraction process corresponding to traditional brewing. The material was steeped three times in water at 75 °C, with a contact time of at least 5 min for each cycle.

The approximate structural composition of the yerba mate biomass used is as follows: cellulose 32–39%, hemicellulose 26–32%, lignin 25–30%, and proteins 4–12% [29,30,31,32,33,34].

### 2.2. Dyes

**Figure 1:**
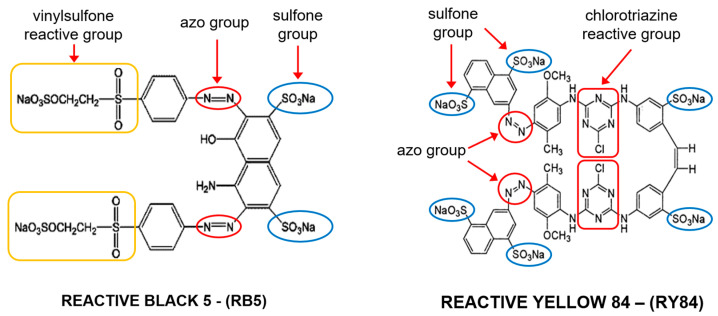
Chemical structure of RB5 and RY84 dyes [35].

Two commercial anionic reactive dyes were used in the study: Reactive Black 5 (RB5) and Reactive Yellow 84 (RY84) (Figure 1). Both compounds were supplied by Boruta-Zachem S.A. (Zgierz, Poland) and used without further purification. Reactive Black 5 is a bifunctional diazo dye containing two reactive vinyl sulfone groups, which account for its high reactivity and water solubility. Reactive Yellow 84 is a polyreactive azo dye with two reactive chlorotriazine groups, which provide high affinity for the hydroxyl groups of the material. The fundamental physicochemical properties of the adsorbates are summarized in Table 1.

### 2.3. Chemical Reagents

The high-purity reagents (analytical grade, ≥99% purity) required for this study were obtained from Sigma-Aldrich, located in St. Louis, MO, USA. A detailed list of these materials and their specific applications is presented in Table 2.

### 2.4. Laboratory Equipment

The specific analytical instruments and laboratory equipment required for this study are detailed in Table 3.

## 3. Methodology

### 3.1. Preparation of Adsorbent Based on Spent Yerba Mate (YMs)

The raw material for adsorbent preparation was spent yerba mate waste obtained after the brewing process (Section 2.1). To remove residual soluble substances and natural pigments that could interfere with the reliability of spectrophotometric measurements, the biomass was intensively rinsed on a laboratory sieve with deionized water. Rinsing continued until the effluent was completely colorless. Following the washing step, the biomass was dehydrated in a laboratory oven at 105 °C. The resulting dry material was then fractionated using 3 mm and 1 mm mesh screens to separate the specific 1–3 mm size range. The 1–3 mm fraction was selected to ensure representative sampling of the heterogeneous biomass (leaves and stems) and to provide optimal conditions for subsequent separation of the adsorbent from the aqueous phase, while also minimizing energy consumption during material preparation. The unmodified spent yerba mate bioadsorbent (YMs) was preserved in hermetically sealed polypropylene containers at 25 °C. This storage protocol was employed to safeguard the physicochemical stability of the biomass until the commencement of the sorption experiments.

### 3.2. Preparation of Aminated Adsorbent Based on Spent Yerba Mate (AYMs)

The YMs adsorbent underwent a two-step chemical modification. The selection of reaction parameters, including reagent concentrations, temperatures, and reaction times, was based on previously established and optimized protocols for the amination of lignocellulosic materials [24,25,26]. First, 20 g of dry YMs was placed in a 500 mL Erlenmeyer flask and subjected to 10 min of alkaline activation using 40 mL of 5% NaOH solution. This step was intended to deprotonate the hydroxyl groups and enhance their nucleophilic reactivity. Next, 200 g of epichlorohydrin (99%) was added. The flask was covered with parafilm and shaken for 12 h in a water bath at 60 °C, 100 rpm, and 30 mm amplitude. After epoxidation, the biomass was washed sequentially with isopropanol and thoroughly with deionized water to remove unreacted reagents.

**Figure 2:**
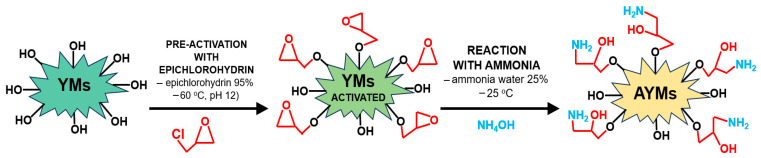
Simplified scheme of the yerba mate biomass modification.

Following the epoxidation phase, the yerba mate biomass was transferred to a 500 mL Erlenmeyer flask for treatment with 200 mL of a 25% aqueous ammonia solution. This amination stage was maintained for 12 h at a regulated temperature of 25 °C, utilizing the same agitation settings as the previous step. The final product, designated as AYMs (aminated yerba mate biomass), was thoroughly washed with deionized water to ensure the removal of residual ammonia, as confirmed by a stable effluent pH below 7.5 and the total absence of odor. Finally, AYMs was dried at 105 °C and stored in airtight polypropylene vessels at ambient temperature. The modification pathway is illustrated in Figure 2.

### 3.3. Adsorbent Characterization Research

#### 3.3.1. Fourier-Transform Infrared Spectroscopy (FTIR)

Fourier-transform infrared (FTIR) spectroscopy was performed to verify the successful chemical transformation and identify the surface functional groups of both YMs and AYMs. For all measurements, a FT/IR-4700LE spectrometer (JASCO International, Tokyo, Japan) was utilized, which was configured with a single-reflection diamond Attenuated Total Reflection (ATR) unit. Absorbance data were captured across a wavenumber range of 4000 to 400 cm^−1^ at a spectral resolution of 1 cm^−1^. To ensure high spectral quality and optimize the signal-to-noise ratio, each reported spectrum was generated by averaging 64 individual scans. Measurements were performed at room temperature directly on solid samples, without KBr pellet preparation, preserving the surface structure of the studied materials.

#### 3.3.2. Specific Surface Area Measurement (BET)

To evaluate the textural characteristics of the YMs and AYMs materials, low-temperature nitrogen adsorption–desorption measurements (77 K) were conducted using an automated ASAP 2020 analyzer (Micromeritics, Norcross, GA, USA). To guarantee high measurement accuracy, all samples underwent a 4 h degassing phase at 100 °C under vacuum to remove adsorbed water and volatile impurities. The specific surface area was established through the multipoint Brunauer–Emmett–Teller (BET) equation. Furthermore, the total pore volume and mean pore diameter were calculated based on the volume of nitrogen adsorbed at a relative pressure p/p_0_ of roughly 0.99.

#### 3.3.3. Elemental Analysis (C, N)

The carbon (C) and nitrogen (N) content in the studied adsorbents was determined using a Flash 2000 elemental analyzer (Thermo Fisher Scientific, Waltham, MA, USA). Before measurement, the materials were dried and homogenized in an agate mortar. Samples weighing between 3 and 5 mg were placed in tin capsules. The determination involved a dynamic flash combustion process in a redox furnace featuring integrated electronic temperature management. The device operated in C/N mode, providing a measurement range of 0.01–100.00% and sensitivity at the level of several parts per million (ppm).

#### 3.3.4. Determination of the Point of Zero Charge (pH_PZC_)

The surface acid-base properties and the point of zero charge (pH_PZC_) for both YMs and AYMs were evaluated via the pH drift technique. The experimental setup consisted of ten individual flasks, each filled with 100 mL of a 0.01 M NaCl background electrolyte. Standardized 0.1 M HCl and NaOH solutions were utilized to adjust the initial pH (pH_0_) across a range of 2.0 to 11.0 at 1.0 pH unit intervals. A fixed sorbent dosage of 10 g/L (1.00 g per vessel) was subsequently introduced into each flask. The resulting suspensions were agitated at 200 rpm for 12 h using magnetic stirrers at ambient temperature to allow the system to reach chemical equilibrium. Afterward, the equilibrium pH (pH_e_) of each solution was recorded. The pH_PZC_ was determined by identifying the zero-crossing on a plot of ΔpH (pH_e_ − pH_0_) versus the initial pH.

### 3.4. Studies on the Effect of pH on Dye Adsorption Efficiency

To determine how the initial solution pH influences dye removal performance, batch-mode experiments were conducted. A precision balance was used to weigh 5.0 g of the dry sorbent (YMs or AYMs) into 600 mL beakers, ensuring a constant adsorbent concentration of 10 g·L^−1^. Subsequently, 500 mL of dye solution (50 mg·L^−1^) was introduced into each vessel, with the pH values modulated between 2.0 and 11.0 using standardized NaOH and HCl solutions. The mixtures were subjected to intensive agitation for 2 h at room temperature using a multi-position magnetic stirrer at 200 rpm, utilizing 40 × 8 mm stir bars. Upon completion of the contact time, 10 mL aliquots were extracted from each beaker via an automatic pipette and transferred to test tubes for subsequent concentration analysis. To track changes in solution acidity during the process, the final pH values were recorded immediately after the sorption phase ended.

### 3.5. Dye Adsorption Kinetics Studies

Kinetic studies were conducted to determine the rate of dye binding on the adsorbent surfaces and to identify the mechanism controlling the adsorption process. The experiments were performed in 1000 mL reaction vessels, each containing 10.00 g (dry weight) of the respective adsorbent (YMs or AYMs) and 1000 mL of dye solution with an initial concentration of either 50 or 500 mg·L^−1^. This large-scale approach was specifically chosen to maintain a constant adsorbent mass-to-solution volume ratio (m:V) throughout the sampling process; since the total volume removed for analysis did not exceed 3% of the initial volume, the associated error could be neglected. Furthermore, using a larger mass of the 1–3 mm biomass fraction ensured a statistically representative sampling of the leaves and stems and provided better hydrodynamic conditions, reducing the risk of mechanical attrition of the particles during stirring. The studies were carried out at pH 3.0, identified as the optimal value for anionic dye adsorption on both tested materials (the justification for this pH selection is provided in Section 4.2). The adsorbent-solution mixtures were continuously stirred using a multi-position magnetic stirrer (200 rpm) and Teflon-coated magnetic stir bars (50 × 8 mm). At precisely defined time intervals (0, 10, 20, 30, 45, 60, 90, 120, 150, 180, 210, and 240 min), 2 mL samples were collected using automatic pipettes and placed into pre-labeled test tubes.

### 3.6. Maximum Adsorption Capacity Studies

To determine the adsorption isotherms and the maximum adsorption capacity of the YMs and AYMs materials for the tested dyes, a series of batch studies were conducted over a wide range of initial concentrations. In 500 mL beakers, 5.0 g (dry weight) of each adsorbent was weighed, maintaining a constant adsorbent mass-to-solution volume ratio of 10 g·L^−1^. Then, 500 mL of dye solutions with initial concentrations ranging from 10 to 1000 mg·L^−1^ were added. The dye solutions were adjusted to pH 3.0 (justification in Section 4.2). The systems were then agitated using a multi-position magnetic stirrer (200 rpm, stir bar dimensions: 40 × 8 mm) for a duration sufficient to reach thermodynamic equilibrium (determined based on Section 3.5). After this interval, 10 mL aliquots were extracted from the beakers using an automatic pipette and transferred to pre-prepared test tubes for concentration measurement.

### 3.7. Analytical Procedures and General Experimental Conditions

All process studies (pH effect, kinetics, and isotherms) were conducted at a constant ambient temperature of 25 °C. Two reactive dyes, Reactive Black 5 (RB5) and Reactive Yellow 84 (RY84), were used as model pollutants in the adsorption studies. Working solutions of specified concentrations were prepared for each experiment using deionized water. The pH of the solutions was adjusted to the desired values using standard 1 M HCl and 1 M NaOH solutions, with continuous monitoring by a pH meter.

The adsorbents (YMs and AYMs) were weighed using a precision balance with an accuracy of 0.001 g, maintaining a constant adsorbent dosage of 10 g·L^−1^ across all experimental series. This dosage was selected to ensure a representative sampling of the heterogeneous biomass particles (1–3 mm fraction) and to achieve measurable changes in dye concentration, especially for the raw YMs which possesses a relatively low specific surface area. Furthermore, it ensured representative sampling of the heterogeneous biomass, which had a relatively large particle size fraction of 1–3 mm. To ensure optimal system hydrodynamics and eliminate diffusion resistance in the boundary layer, the solutions were stirred at a minimum rotational speed that ensured uniform adsorbent distribution throughout the liquid volume.

Dye concentrations were determined spectrophotometrically using a UV-VIS spectrophotometer and quartz cuvettes with a 10 mm optical path length. Measurements were performed at the maximum absorbance wavelengths (λ_max_) of 600 nm for RB5 and 356 nm for RY84. Dye concentrations in the samples were calculated based on calibration curves prepared for RB5 and RY84 in the conc. range of 0–50 mg·L^−1^. Samples with concentrations exceeding this range were diluted with deionized water before measurement.

Particular attention was given to eliminating errors resulting from the potential leaching of natural pigments (e.g., tannins, polyphenols) from the biomass into the solution. For this purpose, a parallel series of control experiments (without synthetic dye) was conducted for each adsorbent. The resulting solutions were used as a reference (blank) during spectrophotometer calibration before the actual measurements. Each experimental series was conducted in three independent trials to ensure reproducibility, and the data reported herein reflect the calculated arithmetic means.

### 3.8. Computational Methods and Mathematical Modeling

The specific equilibrium sorption capacity for the YMs and AYMs materials was computed according to the mass balance principle expressed in Equation (1):

(1) $$q_{e}=(C_{0}-C_{e})\times \frac{V}{m}$$

q_e_—equilibrium adsorption capacity [mg·g^−1^].

C_0_—initial dye concentration in the solution [mg·L^−1^].

C_e_—equilibrium (final) dye concentration in the solution [mg·L^−1^].

V—solution volume [L].

m—mass of the dry adsorbent [g].

To describe the adsorption kinetics, the experimental data were fitted using the pseudo-first-order (PFO) (2), pseudo-second-order (PSO) (3), and intraparticle diffusion (4) models:

(2) $$q_{t}=q_{e}\times (1-e^{\left(-k_{1}\times t\right)})$$

(3) $$q_{t}=\frac{\left(k_{2}\times {q_{e}}^{2}\times t\right)}{\left(1+k_{2}\times q_{e}\times t\right)}$$

(4) $$q_{t}=k_{id}\times t^{0.5}$$

q_t_—amount of dye adsorbed at time t [mg·g^−1^].

q_e_—amount of dye adsorbed at equilibrium [mg·g^−1^].

t—adsorption time [min].

k_1_—PFO rate constant [1·min^−1^].

k_2_—PSO rate constant [g·mg^−1^·min^−1^].

k_id_—intraparticle diffusion rate constant [mg·(g·min^0.5^)^−1^].

The characterization of adsorption equilibrium and the assessment of the maximum capacity of YMs and AYMs were performed using three models: the single-site Langmuir isotherm (5), the dual-site Langmuir isotherm (6), and the Freundlich model (7):

(5) $$q_{e}=\frac{\left(Q_{max}\times K_{L}\times C_{e}\right)}{\left(1+K_{L}\times C_{e}\right)}$$

(6) $$q_{e}=\frac{\left(b_{1}\times K_{1}\times C_{e}\right)}{\left(1+K_{1}\times C_{e}\right)}+\frac{\left(b_{2}\times K_{2}\times C_{e}\right)}{\left(1+K_{2}\times C_{e}\right)}$$

(7) $$q_{e}=K_{F}\times C^{\frac{1}{n}}$$

q_e_—equilibrium adsorption capacity [mg·g^−1^].

Q_max_—maximum adsorption capacity in the Langmuir model [mg·g^−1^].

b_1_,b_2_—maximum adsorption capacity for the first and second types of active sites, respectively [mg·g^−1^].

K_L_—Langmuir equilibrium constant related to adsorption energy [L·mg^−1^].

K_1_,K_2—_adsorption affinity constants for the respective sites [L·mg^−1^].

K_F_—equilibrium adsorption constant in the Freundlich model [-].

n—surface heterogeneity parameter.

C_e_—concentration of dye remaining in the solution at equilibrium [mg·gL^−1^].

## 4. Results and Discussion

### 4.1. Characterization of the Tested Adsorbents

#### 4.1.1. FTIR Spectra Analysis of YMs and AYMs

**Figure 3:**
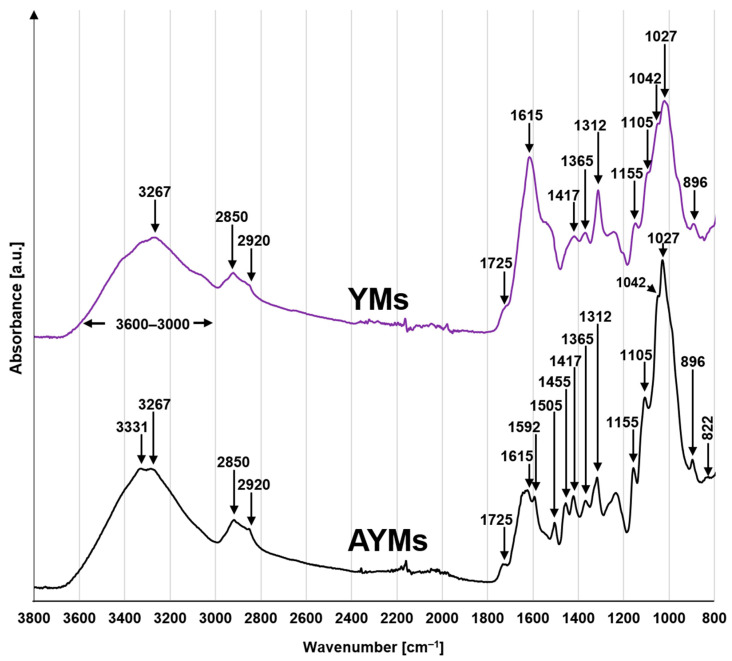
FTIR spectra for YMs and AYMs.

FTIR spectra analysis was performed to identify key functional groups on the surface of yerba mate biomass and to confirm the chemical changes resulting from its amination (Figure 3).

The infrared profile of the untreated yerba mate waste (YMs) displays spectral features consistent with the complex structure of lignocellulosic biomass. A prominent, wide absorption envelope spanning 3600–3000 cm^−1^ (centered at 3267 cm^−1^) is assigned to the stretching vibrations of hydroxyl (–OH) moieties inherent to lignin and cellulose [36]. Furthermore, the signals identified at 2920 and 2850 cm^−1^ arise from the asymmetric and symmetric C–H stretching vibrations associated with aliphatic groups [37]. The presence of hemicelluloses and lignin is confirmed by the peak at 1725 cm^−1^, assigned to the stretching vibrations of carbonyl groups (C=O) in ester bonds and free carboxylic groups [38]. The presence of these –COOH groups determines the acidic character of the biomass surface, which is reflected in the low pH_PZC_ value (Section 4.3). Bands in the range of 1615–1312 cm^−1^ (1615, 1417, 1365, and 1312 cm^−1^) originate from the skeletal vibrations of aromatic rings and the deformation vibrations of –OH and C–H groups [39]. Additionally, the intense set of peaks in the 1155–1027 cm^−1^ region (1155, 1105, 1042, and 1027 cm^−1^) is typical for the stretching vibrations of C–O and C–O–C bonds in the glycosidic structure of cellulose, further confirmed by the peak at 896 cm^−1^, which indicates the presence of β-glycosidic bonds [40,41].

In the spectrum of aminated spent yerba mate (AYMs), a new peak appears at 3331 cm^−1^, overlapping with the hydroxyl group band and indicating the presence of N–H stretching vibrations from primary amino groups (–NH_2_) [42] (Figure 3). Key evidence for the introduction of nitrogen into the biomass structure is the appearance of a distinct peak at 1592 cm^−1^, corresponding to N–H deformation vibrations [43]. Additionally, new bands are observed in the aminated material at 1505 cm^−1^ and 1455 cm^−1^, which can be assigned to C–N stretching vibrations [44] and –CH_2_ deformation vibrations [45] originating from the linker introduced by epichlorohydrin. The presence of a new signal at 822 cm^−1^ suggests changes in aromatic ring substitution [46] or the presence of deformation vibrations from new nitrogen-containing groups. The peaks described above confirm that the amination process of YMs successfully introduced amino groups into the adsorbent structure (AYMs).

#### 4.1.2. Elemental Analysis (C, N Content)

To quantitatively confirm the effectiveness of the chemical modification and determine changes in the mass composition of the bioadsorbents, an elemental analysis of the materials was conducted before and after the amination process (Table 4).

The elemental composition analysis of the unmodified yerba mate biomass (YMs) revealed a nitrogen content of 1.82%, which is typical for plant-derived materials and results from the presence of residual proteins and alkaloids (including caffeine) naturally occurring in *Ilex paraguariensis* leaves. Key evidence of successful functionalization is the clear increase in the nitrogen mass fraction in the AYMs sample, which reached 2.23%. This represents a relative enrichment of the matrix by 22.5% compared to the raw material. This increase is a direct consequence of the chemical modification of the adsorbent, which introduced primary amino groups (-NH_2_) into the structure of the lignocellulosic polymers. It is also worth noting the change in the N/C ratio, which increased from 0.0393 (YMs) to 0.0454 (AYMs). The increase in this parameter clearly indicates a denser packing of nitrogen centers within the carbon skeleton of the adsorbent. These results are consistent with the FTIR analysis, particularly regarding the appearance of new deformation vibration bands for N–H bonds (approx. 1592 cm^−1^) and stretching vibrations for C-N bonds (approx. 1505 cm^−1^).

#### 4.1.3. Point of Zero Charge (pH_PZC_)

**Figure 4:**
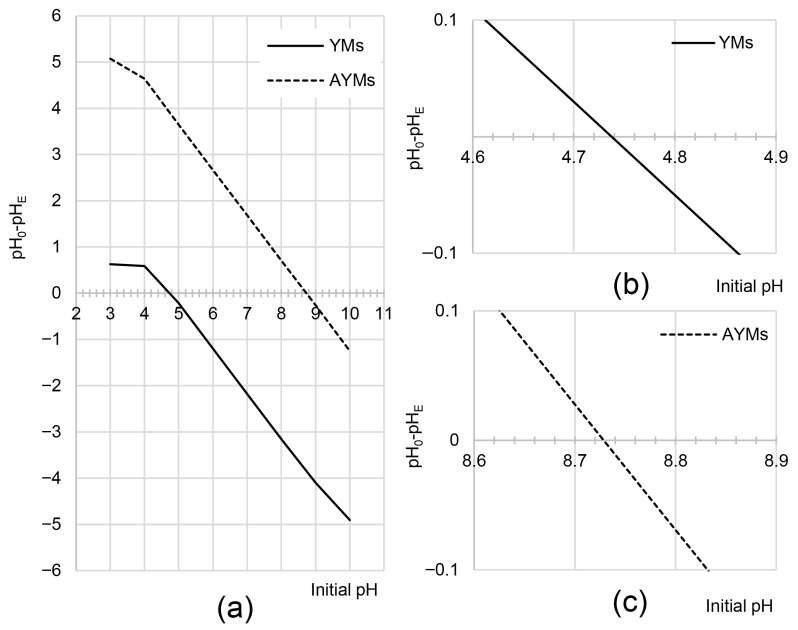
(**a**) pH_PZC_ of YMs and AYMs determined by the drift method; (**b**) inset for YMs; (**c**) inset for AYMs. Temp. 25 °C.

Determining the point of zero charge (pH_PZC_) is critically important for understanding the adsorption mechanism, as this parameter defines the solution pH at which the surface charge of the adsorbent is electrically neutral. The results determined by the pH drift method for YMs and AYMs are presented in Figure 4.

The pH_PZC_ value for YMs was 4.74, indicating an acidic surface character. This is typical for unprocessed plant materials, which are dominated by acidic functional groups such as carboxylic groups (-COOH) present in pectins and hemicelluloses, and hydroxyl groups (-OH) in the lignin structure. As a result of the chemical modification, the pH_PZC_ value for the AYMs material shifted significantly toward alkaline values, reaching 8.73. Such a substantial increase (by approximately 4 pH units) provides definitive confirmation of the successful introduction of basic amino groups into the adsorbent structure. This result correlates directly with the elemental analysis (increase in N content) and the FTIR spectra (appearance of N-H and C-N bands). The high pH_PZC_ value for AYMs predicts that this adsorbent will exhibit significantly higher adsorption efficiency toward anionic dyes compared to YMs, particularly in slightly acidic and neutral environments.

#### 4.1.4. BET Surface Area and Porosity

The textural parameters of unmodified spent yerba mate biomass (YMs) and its aminated form (AYMs), determined by the BET (Brunauer–Emmett–Teller) method, are summarized in Table 5. The textural parameters were determined using N_2_ adsorption; however, due to the relatively low surface area of the biomass, the results are reported with reduced decimal precision to avoid overestimating the analytical certainty of the method.

Analysis of the results indicates that both materials have a relatively low specific surface area (BET < 2 m^2^·g^−1^), which is typical for natural lignocellulosic materials that have not undergone intensive thermal activation. According to the IUPAC classification, the average pore diameter (Dp) values in the range of 2.2–2.3 nm classify the studied adsorbents as mesoporous materials. For the AYM, a slight decrease was observed in all measured textural parameters compared to the raw material (YMs). Although these differences are within the range of measurement uncertainty, the systematic reduction in the mean values of BET, Vp, and Dp suggests successful chemical modification. These changes can be attributed to the deposition of epichlorohydrin molecules and the introduction of amino groups on the surface and within the pores of the biomass, leading to partial narrowing or blockage. Maintaining the textural parameters at a similar level is a significant research finding, demonstrating that the applied two-step chemical modification did not destructively affect the structural skeleton of the biomass.

It is noteworthy that despite the low specific surface area (<2.2 m^2^·g^−1^), AYMs exhibited remarkably high adsorption capacities. This suggests that the adsorption process is not primarily driven by physical pore-filling or the extent of the internal surface area, but rather by the density of functional groups on the surface. The slight decrease in BET surface area and pore volume after amination further confirms the successful attachment of epichlorohydrin linkers and amino groups, which partially occupy or obstruct the original pores of the YMs matrix. Consequently, the performance of AYMs, as further detailed in Section 4.4, is governed by a “chemistry-over-texture” mechanism, where chemical affinity outplays physical adsorption.

### 4.2. Effect of pH on Dye Adsorption Efficiency

**Figure 5:**
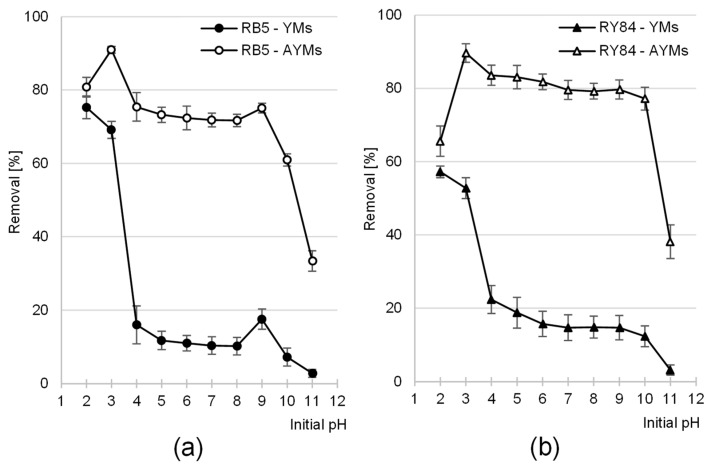
Effect of pH on the adsorption efficiency of (**a**) RB5 and (**b**) RY84 on YMs and AYMs (mean of 3 measurements ± range). Initial dye concentration = 50 mg·L^−1^. Temp. 25 °C.

The adsorption of RB5 and RY84 on YMs was most effective at pH 2, while for AYMs, the optimum was observed at pH 3 (Figure 5). Increasing the system’s pH led to a decrease in dye binding efficiency. However, fundamental differences were observed in the performance characteristics of the raw and aminated materials. For unmodified yerba mate biomass, a sharp decline in efficiency occurred as the pH increased from 3 to 4.

The aminated yerba mate biomass exhibited a different performance profile, characterized by a relatively high and stable level of dye removal across a broad solution pH range (pH 3 to pH 10). A sharp drop in the AYMs adsorption curve was observed only after exceeding pH 10. An interesting phenomenon for the aminated adsorbent was the slight decrease in the adsorption of both dyes when the pH dropped from 3 to 2, which did not occur in the YMs experimental series. Furthermore, specifically for the RB5 dye, a slight local increase in efficiency was recorded at pH 9 for both materials, which was not observed in the series with the RY84 dye.

Similar dye adsorption results, manifested by a decrease in adsorption efficiency with increasing system pH, have also been reported in studies on RB5 adsorption onto goldenrod biomass [27] and wheat straw [47], as well as in studies on RY84 binding onto compost [48] and cotton fibers [24].

The observed differences in adsorption performance can be explained by changes in the surface chemistry of the materials, considering the pH_PZC_ values and the degree of ionization of functional groups. The functional groups of YMs potentially involved in the process included hydroxyl and carboxyl groups, as well as a few naturally occurring amino groups. AYMs was characterized by a significantly higher concentration of primary amino groups introduced during the modification process. The RB5 and RY84 dyes owe their anionic character to the presence of acidic sulfonic groups (–SO_3_^−^).

In an acidic environment (pH 2–3), protons were attached to the functional groups of the adsorbents. Consequently, at pH 2–3, the adsorbent surface gained a net positive charge (or lost its negative charge), which favored the adsorption of anionic dyes through electrostatic interactions.

An increase in the system pH (and thus a decreasing concentration of hydronium ions) resulted in reduced protonation efficiency of the functional groups. At pH > 3, most carboxyl groups remained in their ionized form. As a result, increasing the initial pH from 3 to 4 led to a dramatic reduction in the surface charge of YMs, explaining the drop in adsorption efficiency in this range (Figure 5). At pH > 5, YMs already possessed a net negative surface charge (pH_PZC_ YMs = 4.74), which explains the low efficiency of anionic dye adsorption.

The AYMs adsorbent differed from YMs by its high adsorption efficiency across a broad pH range (3–10). This was due to the high density of introduced amino groups, which undergo protonation over a much wider pH range:

−NH_2_ + H_2_O → −NH_3_^+^ + OH^−^

Consequently, AYMs was able to effectively bind dyes even at pH 9–10, which is above its point of zero charge (pH_PZC_ AYMs = 8.73). The efficiency significantly decreased only at pH > 10, when the amino groups underwent deprotonation. This robust performance is a direct consequence of the chemical transformation confirmed by the FTIR spectra (appearance of N–H and C–N bands, Figure 3) and the 22.5% increase in nitrogen content (Table 4). While raw YMs relies on its native, pH-sensitive structure, the nitrogen-enriched matrix of AYMs maintains a stable positive charge across a wider range of conditions, establishing a clear link between the targeted chemical modification and the expanded operational window of the adsorbent.

The adsorption of dyes onto YMs and AYMs appears to involve a multifaceted interaction profile, indicating that electrostatic attraction between ionized surface moieties and the adsorbates is not the solitary driver of the process. The binding of RB5 and RY84 on the tested plant biomass may also result, to some extent, from the formation of hydrogen bonds or π–π interactions between the aromatic frameworks of the lignin matrix and the benzenoid rings of the dye molecules.

The decrease in AYMs efficiency at pH 2 is likely due to the high concentration of chloride ions (Cl^−^) originating from the HCl used for pH adjustment. At pH 2, their concentration is 10 times higher than at pH 3; therefore, Cl^−^ anions effectively compete with large dye molecules for the protonated adsorption sites. A similar result, manifested by lower anionic dye adsorption efficiency at pH 2 than at pH 3, is frequently observed in studies on the adsorption properties of amino-rich adsorbents (e.g., aminated plant biomass [25,26,27,47] chitosan adsorbents [49]).

The minor enhancement in RB5 uptake recorded at pH 9 is likely associated with the amino (–NH_2_) moieties present in the dye’s molecular framework. Despite the negative charge of the adsorbent surfaces, the presence of the amino group in the RB5 structure could generate local interactions, thereby increasing the probability of adsorption, as also noted in the literature [24,49,50].

**Figure 6:**
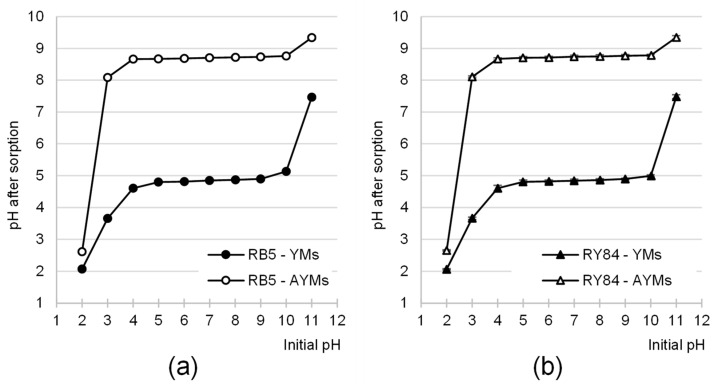
Changes in solution pH values during the adsorption of (**a**) RB5 and (**b**) RY84 on YMs and AYMs (mean of 3 measurements ± range). Initial dye concentration = 50 mg·L^−1^. Temp. 25 °C.

AYMs and YMs altered the pH of the dye solutions during adsorption (Figure 6). These changes resulted from the pH_PZC_ values of the tested adsorbents. As a result, the system pH always gravitated toward the pH_PZC_ values of the adsorbents, i.e., pH 4.74 for YMs and 8.73 for AYMs.

Considering practical aspects and the fact that at pH 3.0 both adsorbents exhibit high and comparable operational stability, pH 3.0 was selected as the optimal value for further kinetic and equilibrium studies.

### 4.3. Dye Adsorption Kinetics

The adsorption equilibrium time is a key technological parameter for assessing the rate of the decolorization process. For the unmodified biomass (YMs), the time required to reach equilibrium was relatively long (150–210 min) and depended on both the type of adsorbate and its initial concentration (Table 6). The slightly shorter time observed for RY84 compared to RB5 is likely due to differences in chemical affinity. Despite its higher molar mass, the triazine rings in the RY84 structure facilitate stronger hydrogen bonding and π–π interactions with the lignin components of the yerba mate matrix, allowing for faster anchoring on the surface.

The extension of equilibrium time with increasing initial dye concentration on YMs resulted from the depletion of easily accessible external active sites (Table 6). This forced the dye molecules to penetrate deeper layers of the lignocellulosic structure—a significantly slower process that becomes the rate-limiting step for reaching full thermodynamic equilibrium.

The introduction of amino groups (AYMs) significantly reduced the equilibrium time to a constant of 120 min, regardless of dye type or concentration (Table 6). This indicates a shift in the process mechanism; the high density of –NH_3_^+^ groups generates strong electrostatic attraction, which forces rapid transport of dye molecules to the active sites. The constant time of 120 min suggests that for AYMs, the process is controlled by rapid surface ion exchange, and the material possesses a sufficiently large number of active sites to prevent surface clogging even at high dye concentrations.

**Figure 7:**
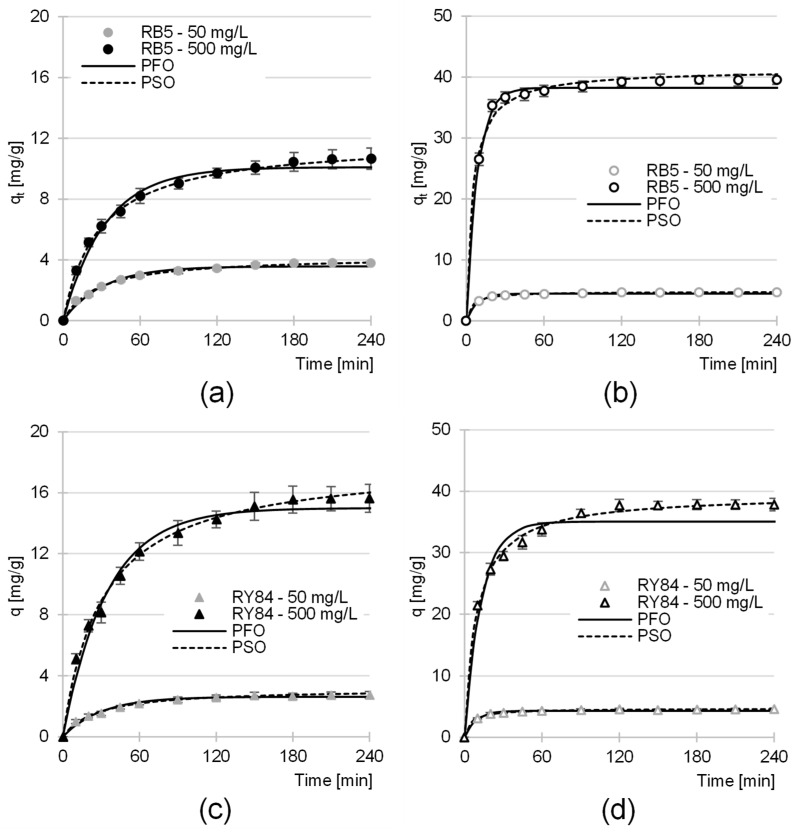
Adsorption kinetics of (**a**) RB5 on YMs, (**b**) RB5 on AYMs, (**c**) RY84 on YMs, and (**d**) RY84 on AYMs (mean of 3 measurements plus range). Pseudo-first-order and pseudo-second-order models. Temperature 25 °C.

**Figure 8:**
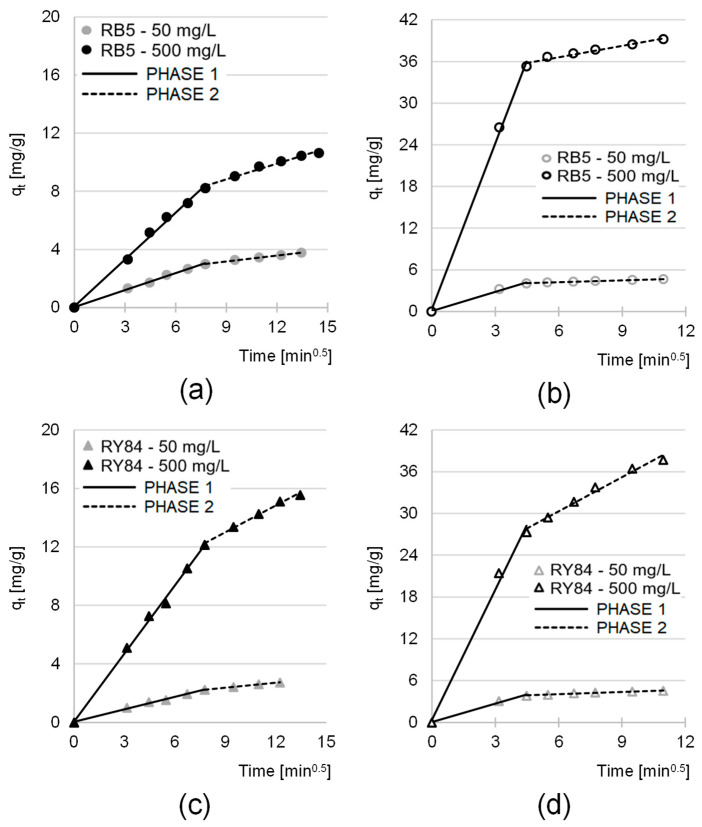
Intraparticle diffusion model for the adsorption of (**a**) RB5 on YMs, (**b**) RB5 on AYMs, (**c**) RY84 on YMs, and (**d**) RY84 on AYMs (mean of 3 measurements plus range). Temp. 25 °C.

The experimental data were fitted using pseudo-first-order (PFO) and pseudo-second-order (PSO) models (Figure 7, Table 6), as well as the intraparticle diffusion model (Figure 8, Table 7), to identify the mechanism controlling adsorption. Based on the kinetic parameters (Table 6), the PSO model yielded the highest coefficients of determination (R^2^ > 0.99) and q_e,cal_ values that closely matched the experimental data. The superior fit of the PSO model confirms that the process is governed by chemisorption for both dyes on YMs and AYMs.

Analysis of the intraparticle diffusion model (Table 7) indicated that the process proceeded in two distinct phases. Phase I, involving dye diffusion to the adsorbent surface, was exceptionally intense for AYMs (20 min) compared to YMs (60 min), highlighting the impact of strong electrostatic attraction introduced by amination. After surface saturation, the process transitioned into a slower Phase II (90–150 min), during which dyes penetrated the deeper layers of the matrix. The linear plots for the first adsorption phase passed close to the origin (Figure 8), suggesting that mass transfer resistance in the boundary layer was not the rate-limiting factor, thereby confirming optimal hydrodynamic conditions during the experiments.

The diffusion rate constants (k_d1_ and k_d2_) showed a strong correlation with the initial dye concentration (Table 7). Significant increases in these constants with higher C_0_ values result from the increased concentration gradient, which facilitates the penetration of dye molecules into the adsorbent structure. Although equilibrium was established within 120–210 min, a unified contact time of 240 min was selected for subsequent adsorption isotherm studies to ensure complete saturation of active sites in all analyzed systems.

### 4.4. Maximum Adsorption Capacity

**Figure 9:**
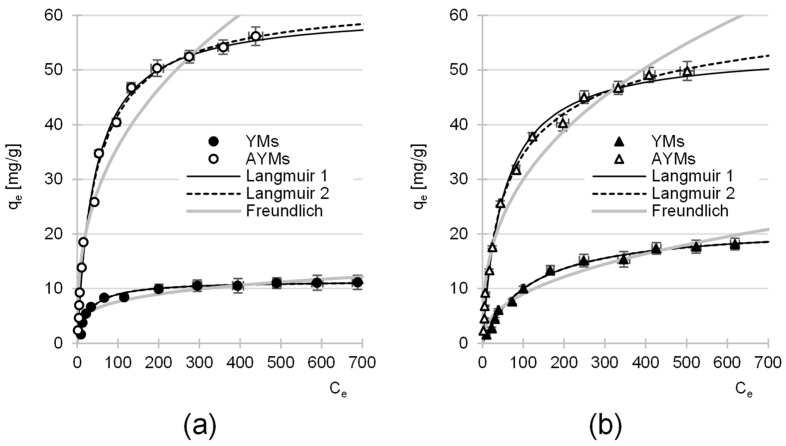
Isotherm of adsorption of: (**a**) RB5, (**b**) RY84 on YMs and AYMs (mean of 3 measurements plus range). Temp. 25 °C.

The experimental data were fitted using the single-site Langmuir and dual-site Langmuir models and the Freundlich model (Figure 9). The isotherm parameters are summarized in Table 8.

In all experimental series, the highest coefficients of determination were obtained for the Langmuir models. The significantly weaker fit of the Freundlich model suggests that the adsorption of RB5 and RY84 dyes on the tested materials is monolayer in nature.

A comparative analysis of the single-site Langmuir and dual-site Langmuir models provides key information about the energetic structure of the surface. For YMs, the parameters determined from both Langmuir models are identical (K_c_ = K_1_ = K_2_), and the R^2^ coefficient remains unchanged. This demonstrates the high energetic homogeneity of the adsorption sites on YMs. For this material, the dye-binding process occurs primarily on a single type of site, most likely protonated hydroxyl groups (-OH_2_^+^).

For AYMs, the dual-site Langmuir model provided a superior fit to the experimental data compared to the single-site model. Notably, the affinity constants K_1_ and K_2_ differ significantly (Table 8), reflecting the energetic heterogeneity of the AYMs surface and providing a quantitative basis for the structure–performance relationship. The sites with higher affinity (K_1_) represent the primary amino groups introduced during the modification process, which act as high-energy centers for strong ionic bonding with the sulfonate groups of the dyes. The presence of these high-affinity sites is directly supported by the nitrogen enrichment confirmed in the elemental analysis and the substantial shift in pH_PZC_. In contrast, the second type of sites (K_2_) corresponds to the inherent lignocellulosic background (such as hydroxyl groups and aromatic structures), which offers lower-energy interactions, including hydrogen bonding or π–π stacking. The synergy between these newly introduced amino groups and the natural functional groups of the biomass explains why AYMs achieves a five-fold increase in adsorption capacity for RB5 and a three-fold increase for RY84 compared to the raw YMs.

The amination of yerba mate biomass resulted in a substantial increase in its maximum adsorption capacity (Q_max_) for anionic dyes. For the RB5 dye, the capacity rose from 11.43 mg·g^−1^ (YMs) to 62.81 mg·g^−1^ (AYMs), representing more than a five-fold increase in efficiency. For the RY84 dye, a nearly three-fold increase was observed, from 21.68 mg·g^−1^ to 61.78 mg·g^−1^.

Finally, it should be acknowledged that the adsorption experiments in this study were conducted at a single constant temperature of 25 °C. Although the equilibrium constants obtained from the Langmuir models (Table 8) provide significant insights into the binding affinity and the energetic heterogeneity of the AYMs surface, the lack of temperature-dependent data limits a comprehensive thermodynamic interpretation. Consequently, standard thermodynamic parameters such as enthalpy change (ΔH°) and entropy change (ΔS°) were not determined in the current work. This limitation is recognized, and future research covering a broader temperature range will be essential to fully elucidate the energetics of the adsorption process.

Beyond the maximum adsorption capacity Q_max_, the performance of an adsorbent can also be evaluated using the partition coefficient (PC) concept, defined as the ratio of q_e_ to C_e_ [51,52]. In the case of AYMs, the high q_e_/C_e_ ratio observed at low equilibrium concentrations (reflected in the steep initial slope of the isotherms in Figure 9) confirms the high affinity of the aminated biomass for reactive dyes. For instance, in the case of RB5, the calculated PC for AYMs at the lowest equilibrium concentration reached 1.63 L·g^−1^, which represents a more than 8-fold increase compared to the raw YMs (0.19 L·g^−1^). A similar trend was observed for RY84, confirming that amination not only increases the total capacity but also significantly enhances the efficiency of dye removal from low-concentration solutions. This is a critical factor for the effective removal of pollutants from dilute industrial effluents, where the adsorbent must operate efficiently even at low residual concentrations.

In summary, the adsorption of RB5 and RY84 onto AYMs is a multi-interaction process. The primary driving force is electrostatic attraction between the positively charged protonated amino groups (–NH_3_^+^) of the modified biomass and the anionic sulfonate groups (–SO_3_^−^) of the dyes. This dominant mechanism is confirmed by the significant shift in pH_PZC_ and the high performance of AYMs across a broad pH range. However, the energetic heterogeneity identified by the dual-site Langmuir model suggests that auxiliary forces, such as hydrogen bonding (between biomass hydroxyl/amino groups and dye heteroatoms) and π–π interactions (between the aromatic rings of lignin and the dye molecules), also contribute to the overall binding process. This synergistic effect, resulting from the successful chemical functionalization of the matrix, accounts for the superior adsorption efficiency of AYMs compared to its raw form.

To objectively assess the application potential of the obtained materials, their maximum adsorption capacities (Q_max_) were compared with literature data for other unconventional adsorbents and activated carbons (Table 9 and Table 10).

A critical evaluation of the data in Table 9 and Table 10 reveals that AYMs occupies a competitive niche between raw biomass and commercial adsorbents. Compared to other raw lignocellulosic materials, the natural structure of *Ilex paraguariensis* shows higher initial adsorption potential than typical agricultural wastes like sunflower husks or cotton fibers, providing an excellent base for modification. While AYMs (62.81 mg·g^−1^) is second only to aminated buckwheat hulls or goldenrod biomass, it surpasses many conventional materials, showing nearly twice the efficiency of activated carbons derived from bamboo or palm shells (Table 9). The primary advantage of AYMs lies in its significantly faster kinetics (120 min) compared to the 12–24 h often required for many commercial carbons. However, a key limitation of AYMs is its likely lower mechanical strength and bulk density compared to granular activated carbons, which may affect its performance in high-pressure column systems. Despite this, the zero-cost precursor and the simplicity of the low-temperature modification process make AYMs an economically superior and more sustainable option for small-scale industrial applications where rapid treatment is prioritized.

Before considering the practical application and waste management of the material, it is essential to synthesize the overall structure–property–performance relationship established in this study. The integration of characterization and adsorption data reveals a clear correlation: the chemical modification successfully introduced amino groups (Structure: FTIR, Elemental Analysis), which fundamentally shifted the surface charge from acidic to basic (Property: pH_PZC_). These changes rendered the physical surface area (BET) secondary to chemical affinity, enabling high-capacity dye removal (Performance: Kinetics, Isotherms). The transition from a single-site adsorption on YMs to a dual-site mechanism on AYMs confirms that the amination created a new, energetically heterogeneous surface, specifically optimized for the remediation of stable anionic pollutants.

The results clearly indicate that AYMs has strong potential to serve as a full-fledged alternative to commercial carbon adsorbents for removing anionic dyes from industrial effluents. The implementation of AYMs on a technical scale is primarily supported by economic factors, as it utilizes a waste raw material with zero market value, drastically reducing precursor acquisition costs. Additionally, the low energy requirements of AYMs production, based on low-temperature chemical modification, align well with the principles of the Circular Economy. The proposed solution offers high adsorption efficiency while maintaining a minimal carbon footprint, providing a significant advantage over traditional, highly energy-intensive activated carbon production.

Unlike high-cost activated carbons, the zero-cost nature of the AYMs precursor makes single-use valorization a more economically viable and environmentally friendly alternative to chemical regeneration, which typically produces secondary liquid waste. The decision to forego traditional regeneration and reusability studies in favor of a single-use valorization strategy is based on a technical-economic analysis of the bioadsorbent’s lifecycle. While regeneration is often mandatory for high-cost synthetic materials, for waste-derived adsorbents like AYMs, the chemical footprint of desorption—requiring concentrated HCl or NaOH—often negates the environmental benefits of using a sustainable precursor. Furthermore, lignocellulosic matrices are susceptible to structural degradation during aggressive chemical cycles, which would lead to a significant loss of adsorption efficiency and mechanical stability.

Consequently, we propose a ‘cascade utilization’ model that aligns with Circular Economy principles. Due to its high lignin (25–30%) and cellulose content, spent AYMs is an ideal candidate for thermal valorization with energy recovery, as the adsorbed dyes do not significantly alter its high calorific value. An alternative is to subject the spent biomass to fermentation for biogas production, as the presence of adsorbed dyes on the lignocellulosic matrix does not significantly inhibit methanogenesis processes [67]. Additionally, the dye-saturated material can be repurposed as a precursor for the production of low-cost activated carbons, or safely immobilized within construction materials (e.g., concrete matrices) to prevent secondary leaching. This multi-directional approach ensures that the material’s lifecycle ends with value-added applications rather than the generation of hazardous saline wastewater from regeneration.

## 5. Conclusions

This study demonstrates that the chemical functionalization of spent yerba mate through amination is an effective strategy for converting low-cost agricultural waste into a high-performance bioadsorbent. The results confirm that the introduced amino groups significantly enhance the adsorption capacity of the modified yerba mate biomass toward reactive dyes (RB5 and RY84).

The scientific contribution of this work lies in providing systematic evidence that the unique chemical matrix of *Ilex paraguariensis*, characterized by a high lignin and protein content, serves as a superior and more robust platform for stable functionalization compared to typical agricultural residues such as cereal husks or stalks described in previous literature. This study establishes that AYMs offers not only higher adsorption capacities but also significantly faster kinetics (reaching equilibrium within 120 min) than many previously reported aminated biosorbents, providing a more efficient and scalable solution for industrial wastewater treatment. Furthermore, its effectiveness at near-neutral pH (up to pH 10 for AYMs) distinguishes it as a versatile material for treating diverse industrial effluents where rapid decolorization is essential.

Despite its high performance, the study identifies certain limitations. The efficiency of AYMs was evaluated in a single-component batch system at a constant temperature, which does not fully account for the complexity of multi-dye industrial effluents or the impact of temperature fluctuations. Furthermore, the lower bulk density of the biomass compared to commercial granular activated carbons may necessitate specialized reactor designs, such as fluidized beds, to prevent significant pressure drops in column systems.

Future research should focus on validating the performance of AYMs in continuous fixed-bed columns and assessing the impact of competing ions and surfactants typically found in textile wastewater. Additionally, a detailed Life Cycle Assessment (LCA) would be beneficial to quantify the environmental advantages of the proposed “single-use and valorization” strategy compared to traditional activated carbon regeneration.

## Figures and Tables

**Table 1:** Characteristics of RB5 and RY84 dyes.

Dye Name	Reactive Black 5 (RB5)	Reactive Yellow 84 (RY84)
Chemical class	Diazo dye	Diazo/Triazine dye
Dye type	Reactive/anionic	Reactive/anionic
Main functional groups	Vinylsulfone, sulfone	Chlorotriazine, sulfone
Molar mass	991 [g/mol]	1628 [g·mol^−1^]
λ_max_	600 [nm]	356 [nm]
Uses	Dyeing cotton, wool, and polyamides in black and dark navy blue	Dyeing cotton and viscose fabrics in bright shades of yellow
Hazards	May cause mutations	May cause mutations; may cause sensitization by inhalation

**Table 2:** Chemical reagents used in these studies.

Reagent	Chemical Formula	Purity	Application
Epichlorohydrin	C_3_H_5_ClO	>99%	Material “activation”
Ammonia (aq. solution)	NH_3_·H_2_O	25%	Amination
Hydrochloric acid	HCl	1 mol·L^−1^	pH adjustment
Sodium hydroxide	NaOH	>99.9%	pH adjustment

**Table 3:** Laboratory equipment used in the research.

Equipment Type	Model	Manufacturer and Origin	Application
pH meter	HI 110	HANNA Instruments, Olsztyn, Poland	pH measurement and adjustment
Shaking water bath	TYP 357	Elpin-Plus, Lubawa, Poland	adsorbent modification (activation, amination)
Multi-position stirrer	MS-53M	JEIO TECH, Daejeon, Republic of Korea	Dye adsorption experiments
Multi-position shaker	SK-71	JEIO TECH, Daejeon, Republic of Korea	Dye adsorption experiments
UV-VIS spectrophotometer	UV-3100 PC	VWR Spectrophotometer, VWR International LLC, Mississauga, ON, Canada	Measurement of dye concentration
FTIR spectrometer	FT/IR-4700LE with ATR attachment	JASCO International, Tokyo, Japan	Recording FTIR spectra of materials
Elemental analyzer	FLASH 2000	Thermo Scientific, Waltham, MA, USA	Carbon and nitrogen content analysis
BET surface area analyzer	ASAP 2020	Micromeritics, Norcross, GA, USA	Surface area and porosity measurement

**Table 4:** Carbon and nitrogen content in YMs and AYMs.

Adsorbent Type	Carbon Content [%]	Nitrogen Content [%]	N/C Ratio
YMs	46.31 ± 0.19	1.82 ± 0.05	0.0393
AYMs	46.93 ± 0.17	2.23 ± 0.08	0.0454

**Table 5:** BET surface area and pore characteristics of YMs and AYMs.

Adsorbent Type	Specific Surface Area BET [m^2^·g^−1^]	Total Pore Volume Vp [cm^3^·g^−1^]	Average Pore Diameter Dp [nm]
YMs	1.7 ± 0.1	0.0019 ± 0.0002	2.3 ± 0.1
AYMs	1.6 ± 0.1	0.0017 ± 0.0002	2.2 ± 0.1

**Table 6:** Kinetic parameters of adsorption of dyes onto YMs and AYMs determined from the pseudo-first order and pseudo-second order models.

Adsorbent	Dye	Dye Conc.	Pseudo-First Order Model			Pseudo-Second Order Model			Exp. Data	Equilibrium Time
			k_1_	q_e, cal._	R^2^	k_2_	q_e, cal_.	R^2^	q_e, exp._	[min]
		[mg·L^−1^]	[1·min^−1^]	[mg·g^−1^]	-	[g·mg^−1^·min^−1^]	[mg·g^−1^]	-	[mg·g^−1^]	
YMs	RB5	50	0.0326	3.58	0.9864	0.0088	4.26	0.9968	3.76	180
		500	0.0305	10.10	0.9866	0.0030	11.88	0.9991	10.63	210
	RY84	50	0.0333	2.63	0.9796	0.0118	3.17	0.9926	2.73	150
		500	0.0289	15.00	0.9839	0.0018	18.08	0.9946	15.55	180
AYMs	RB5	50	0.1232	4.46	0.9948	0.0456	4.80	0.9967	4.65	120
		500	0.1166	38.23	0.9934	0.0050	41.24	0.9977	39.21	120
	RY84	50	0.1149	4.33	0.9921	0.0414	4.70	0.9991	4.52	120
		500	0.0778	35.04	0.9744	0.0028	39.53	0.9958	37.71	120

**Table 7:** Dye diffusion rate constants, determined from the intramolecular diffusion model.

Adsorbent	Dye	Dye Conc.	Phase I			Phase II		
			k_d1_	Time Duration	R^2^	k_d2_	Time Duration	R^2^
		[mg·L^−1^]	[mg·(g·min^0.5^)^−1^]	[min]	-	[mg·(g·min^0.5^)^−1^]	[min]	-
YMs	RB5	50	0.3886	60	0.9964	0.1363	120	0.9945
		500	1.0745	60	0.9950	0.3619	150	0.9770
	RY84	50	0.2831	60	0.9944	0.1133	90	0.9989
		500	1.5515	60	0.9970	0.6118	120	0.9930
AYMs	RB5	50	0.9272	20	0.9918	0.0894	100	0.9857
		500	7.9876	20	0.9978	0.5510	100	0.9595
	RY84	50	0.8784	20	0.9906	0.1070	100	0.9621
		500	6.2291	20	0.9931	1.6355	100	0.9833

**Table 8:** Constants determined from single-site Langmuir, dual-site Langmuir and Freundlich models.

Adsorbent	Dye	Single-Site Langmuir Model			Dual-Site Langmuir Model						Freundlich Model		
		Q_max_	K_c_	R^2^	Q_max_	b_1_	K_1_	b_2_	K_2_	R^2^	K_F_	n	R^2^
		[mg·L^−1^]	[L·mg^−1^]	-	[mg·L^−1^]	[mg·L^−1^]	[L·mg^−1^]	[mg·L^−1^]	[L·mg^−1^]	-	-	-	-
YMs	RB5	11.43	0.036	0.9789	11.43	7.25	0.036	4.18	0.036	0.9789	2.51	4.16	0.8840
	RY84	21.68	0.009	0.9929	21.68	10.86	0.009	10.82	0.009	0.9929	1.24	2.32	0.9525
AYMs	RB5	60.83	0.023	0.9941	62.81	49.47	0.073	13.33	0.016	0.9951	6.48	2.69	0.9580
	RY84	53.76	0.020	0.9960	61.78	39.61	0.030	22.17	0.029	0.9980	5.31	2.66	0.9580

**Table 9:** Comparison of the adsorption capacity of various unconventional lignocellulosic adsorbents and activated carbons for RB5 dye (literature data).

Adsorbent	Adsorption Capacity [mg·g^−1^]	pH of Sorption	Time of Adsorption [min]	Source
Activated carbon Filtrasorb 400 (commercial)	198.00	5.2	720	[53]
Activated carbon (powder)	125.79	2	240	[54]
Aminated Buckwheat hulls	85.18	3	300	[25]
Aminated Goldenrod biomass	71.30	3	120	[27]
Activated carbon modified with SPC	69.90	2	<60	[55]
Aminated Yerba Mate (AYMs)	62.81	3	120	This work
Activated carbon—powdered	58.82	-	-	[56]
Aminated Sunflower seed hulls	51.00	3	240	[26]
Activated carbon from bamboo	39.02	2	60	[57]
Activated carbon from Carob tree	36.90	2	120	[58]
Aminated cotton fibers	36.77	3	120	[24]
Activated carbon from palm shell	25.10	2	300	[59]
Activated carbon from wood (walnut)	19.30	5	400	[60]
Wheat straw	15.70	3	195	[47]
Beech sawdust	13.90	3	1440	[61]
Seed scales of *Eriobotrya japonica*	13.76	3	150	[62]
Cotton seed husks	12.90	2	30	[63]
Yerba Mate (YMs)	11.43	3	210	This work
Newsprint paper	7.12	3	120	[50]
Buckwheat hulls	4.43	3	300	[25]
Sunflower seed hulls	2.89	3	210	[26]
Cotton fibers	2.74	3	240	[24]
Goldenrod biomass	2.32	3	150	[27]
Macadamia seed husks	1.21	3	510	[64]
Sunflower biomass	1.10	2	210	[28]

**Table 10:** Comparison of the adsorption capacity of various unconventional lignocellulosic sorbents and activated carbons for RY84 dye (literature data).

Adsorbent	Adsorption Capacity [mg·g^−1^]	pH of Sorption	Time of Adsorption [min]	Source
Aminated Sunflower seed hulls	63.3	3	240	[26]
Aminated Yerba Mate (AYMs)	61.78	3	120	This work
Aminated Goldenrod biomass	59.28	3	120	[27]
Aminated Cotton fibers	43.34	2	240	[24]
Activated carbon from the Borassus plant	40.0	-	-	[65]
Yerba Mate (YMs)	21.68	3	180	This work
Cotton fibers	15.9	2	240	[24]
Wool	11.0	7	180	[66]
Newsprint paper	10.24	3	150	[50]
Sunflower seed hulls	4.15	2	90	[26]
Goldenrod biomass	2.27	3	180	[27]
Compost	2.2	3	180	[48]

## Data Availability

The original contributions presented in this study are included in the article. Further inquiries can be directed to the corresponding author.
